# Coffee and digestive cancers—what do we know, and where do we go?

**DOI:** 10.1038/s41416-020-0771-4

**Published:** 2020-03-10

**Authors:** Erikka Loftfield, Neal D. Freedman

**Affiliations:** Metabolic Epidemiology Branch, Division of Cancer Epidemiology and Genetics, National Cancer Institute, 9609 Medical Center Dr, Rockville, MD 20850 USA

**Keywords:** Risk factors, Gastrointestinal cancer

## Abstract

Coffee drinking has been inversely associated with liver cancer consistently in prospective studies. Yet, the specific compounds underlying this association, and whether associations vary by preparation method, are unknown. Associations with other sites within the gastrointestinal tract are also unclear. A recent study by Tran et al. leverages the resources of the UK Biobank to begin answering these questions, and suggests important avenues for future work.

## Main

Coffee is one of the most widely consumed beverages worldwide, despite often being considered a guilty pleasure due to long-standing concerns that coffee drinking may increase risks of cancer, cardiovascular disease and other chronic diseases. Yet, the accumulated research literature paints a different picture. In 2015, the U.S. Dietary Guidelines Advisory Committee concluded that drinking up to five cups of coffee per day was compatible with a healthy diet.^1^ Furthermore, in 2016, an International Agency for Research on Cancer (IARC) working group reviewed more than a thousand epidemiological and experimental studies on coffee and cancer.^2^ In their report, coffee was classified as group 3, unclassifiable as to carcinogenicity in humans, with consistent evidence for an inverse association for liver and endometrial cancer. Such conclusions are consistent with those of a recent meta-analysis^3^ that indicated evidence for inverse associations with cancers of the breast, colorectum, endometrium, liver, prostate and skin.

Nevertheless, there remain numerous questions regarding the impact of coffee drinking on health. The chief among them is a lack of understanding as to the mechanisms by which coffee drinking may be related to disease. A cup of coffee is a mixture of hundreds, possibly thousands, of compounds, and the levels of these compounds are altered by a variety of production and preparation processes, including decaffeination, freeze-drying, roasting and brewing. A recent metabolomics study investigating the differences in the chemical composition of commonly consumed coffee types found that instant coffee, with particularly high levels of diketopiperazines and lower levels chlorogenic acids among other differences, markedly differed from other coffee types.^4^ Whether the differences in chemical composition influence coffee-cancer associations is largely unknown, but exploring such exposure heterogeneity may provide important clues about disease aetiology.

Within this context, Tran et al.^5^ examine the association of self-reported coffee drinking with the risk of digestive cancer in the UK Biobank cohort. Despite the large size of the cohort, follow-up remains relatively short, so the numbers of individual cancers and associated statistical power was relatively low for most sites. However, unlike most cohort studies, UK Biobank participants reported whether they typically consumed decaffeinated, instant, ground or other types of coffee. In this way, Tran et al.^5^ provide an important methodological advance over previous studies.

In line with earlier studies, they found that coffee drinking was associated with a lower risk of hepatocellular carcinoma (HCC), the most common type of primary liver cancer, although associations with other types were largely null. Importantly, Tran et al.^5^ found no evidence that the inverse association between coffee drinking and HCC varied by coffee type. Despite small case numbers, these results are consistent with a few prior studies that evaluated brewing type, such as in the ATBC study of Finnish smokers, where inverse associations for coffee drinking with incident liver cancer were observed for both boiled and filtered coffee.^6^ The results for other end points may differ, however. For example, a study on coffee drinking and mortality, which also used data from the UK Biobank cohort, observed stronger inverse associations for ground than for instant coffee intake, particularly for cardiovascular disease mortality.^7^

As follow-up in the UK Biobank continues and the cohort matures, it will be important to update these analyses. In addition, we encourage future cohorts to comprehensively assess coffee type and preparation. To aid in this effort, current versions of dietary assessment tools like the National Cancer Institute’s Dietary History Questionnaire III (DHQ III, https://epi.grants.cancer.gov/dhq3/) and the Automated Self-Administered 24-Hour (ASA24®, https://epi.grants.cancer.gov/asa24/) ask for additional details on the type of coffee consumed.

Studies using biomarkers present another promising line of inquiry in coffee-cancer research. Biomarkers can serve both as objective markers of coffee intake and as indicators of the underlying mechanisms. Coffee drinking has consistently been inversely associated with biomarkers of diabetes and insulin resistance, and inflammation.^3^ Untargeted metabolomics studies have detected dozens of metabolites of both exogenous and endogenous origin that are strongly correlated with coffee drinking as well as incident digestive cancer. In the Prostate, Lung, Colorectal and Ovarian (PLCO) Cancer Screening Trial cohort, biomarkers of caffeine identified using metabolomics were associated with incident colorectal cancer,^8^ whereas associations with self-reported coffee drinking were null. A study on coffee-related metabolites, liver cancer and liver disease mortality in Alpha-Tocopherol, Beta-Carotene Cancer Prevention (ATBC) Study cohort was recently published in the *Journal of National Cancer Institute*.^9^ This study found that metabolites that were positively correlated with coffee drinking, including trigonelline and serotonin, were associated with lower risk of liver cancer and liver disease death, whereas metabolites that were negatively correlated with coffee drinking, including two bile acids, were associated with higher risk of liver cancer and liver disease death. A separate investigation, published in the same journal, identified an association between pre-diagnostic bile acid concentrations and colon cancer risk in the European Prospective Investigation into Cancer and Nutrition (EPIC) cohort.^10^ These observations are supported by experimental models demonstrating that bile acids are critical to the gut–liver–axis, and may contribute to the pathogenesis of liver, colorectal and perhaps other digestive diseases.^11^ These exciting results provide plausible mechanisms linking coffee drinking to gastrointestinal cancer, and merit replication in future studies. The impact of coffee roasting and brewing method on biomarkers of exposure, and response to coffee drinking, remains to be determined; however, we advocate for future studies coupling improved assessment of coffee drinking with biochemical measurements to provide insight into the potential role of coffee drinking on cancer.

Despite long-standing concerns that coffee drinking may cause cancer, the body of evidence suggests that coffee drinking may instead have some beneficial qualities. Yet, the mechanisms by which coffee drinking may affect cancer have not been established. Tran et al.^5^ begin the important process of examining the impact of coffee preparation method. Improved self-report via automated 24-h recalls, and cloud computing coupled with advances in studying the human microbiome, genetic variation and metabolism, as well as high-throughput and reproducible biochemical assays suggest an emerging renaissance in nutritional studies.^12^ Future studies incorporating these new approaches have great potential to clarify the impact of coffee drinking on health, and identify the underlying mechanisms. It is time that we brew a fresh pot and get to work.

## Data Availability

Not applicable.
